# Co-delivery of spiramycin and curcumin nanoemulsions for treating acute (RH strain) and chronic (Tehran strain) toxoplasmosis in BALB/c mice

**DOI:** 10.1007/s00436-025-08588-9

**Published:** 2025-12-02

**Authors:** Saeideh Hashemi-Hafshejani, Amir Amani, Sanaz Jafarpour Azami, Hossein Keshavarz Valian, Mehdi Mohebali, Zahra Rampisheh, Saeedeh Shojaee

**Affiliations:** 1 Department of Parasitology and Mycology, School of Medicine, Iran University of Medical Sciences, Tehran, Iran; 2 Department of Advanced Technologies, School of Medicine, North Khorasan University of Medical Sciences, Bojnurd, Iran; 3 Natural Products and Medicinal Plants Research Center, North Khorasan University of Medical Sciences, Bojnurd, Iran; 4 Department of Medical Parasitology and Mycology, School of Public Health, Tehran University of Medical Sciences, Tehran, Iran; 5 Center for Research of Endemic Parasites of Iran (CREPI), Tehran University of Medical Sciences, Tehran, Iran; 6 Preventive Medicine and Public Health Research Center, Psychosocial Health Research Institute, Department of Community and Family Medicine, School of Medicine, Iran University of Medical Sciences, Tehran, Iran

**Keywords:** Toxoplasmosis, Nanoemulsion, Spiramycin, Curcumin

## Abstract

This study aimed to develop and evaluate a nanoemulsion containing spiramycin and curcumin (NE-CS) to enhance these compounds’ bioavailability and efficacy against acute and chronic toxoplasmosis in mice. Nanoemulsion containing spiramycin and curcumin (NE-CS), spiramycin nanoemulsion (NE-Spi), and a nanoemulsion without the drug (NE) were prepared via spontaneous emulsification and characterized for particle size and zeta potential. Their in vitro activity was tested against tachyzoites of *T. gondii*, RH strain, at different concentrations (250, 125, 62.5, and 31.25 µg/ml) and time points (30, 60, 90, and 120 min). In vivo, BALB/c mice infected with RH and Tehran strains were treated with NE-CS (25 mg/kg/day curcumin + 50 mg/kg/day spiramycin), NE-Spi (50 mg/kg/day), S-Spi, and NE, with infected/untreated mice as controls. Survival time, tachyzoite counts in peritoneal fluid (acute phase), and number and size of tissue cysts (chronic phase) were assessed microscopically. NE-CS significantly decreased the intracellular multiplication of *T. gondii* tachyzoites (5 ± 2.78 × 10^4^) compared with that of the infected/untreated group (3509 ± 435.39 × 10^4^). Oral administration of NE-CS improved therapeutic outcomes in vivo, prolonged survival time in mice (13 days), reduced parasitemia, and decreased both the number (43 ± 5.78) and size (4 ± 1.11 µm) of brain cysts compared to the infected/untreated group (380 ± 17.22 and 112.8 ± 8.28 µm, respectively). This study demonstrated the in vitro efficiency of nanoemulsion co-delivery of curcumin and spiramycin for increasing tachyzoite death, and indicated the possibility of NE-CS and NE-Spi for treating acute and chronic toxoplasmosis in murine models. However, further investigations are needed to confirm their safety and effectiveness across broader models.

**Graphical abstract:**

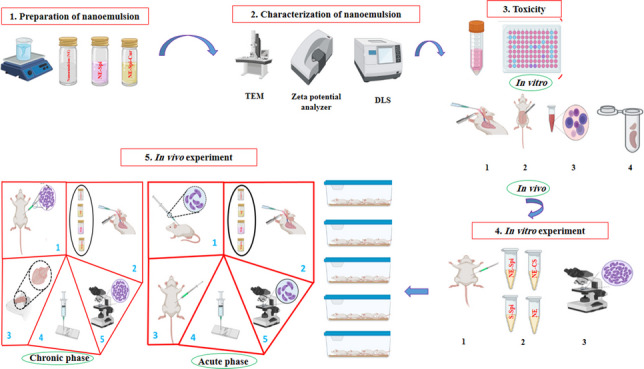

## Introduction

The obligatory intracellular parasite *Toxoplasma gondii* mainly infects warm-blooded animals and humans (Tan et al. 2022). The prevalence of *T. gondii* infection varies from 0 to 100% depending on the geographical area and method of detection (Khosravi et al. 2020)*.* Individuals infected with *T. gondii* usually show no clinical signs in individuals with a healthy immune system (Mihu et al. 2020). However, for immunocompromised HIV-positive patients, cancer patients, and recipients of organ transplants, it can become a potentially fatal illness because of the activation of *T. gondii* cysts in the brain (Rahman et al. 2022). Infections caused by *T. gondii* during pregnancy may result in fetal loss or a variety of congenital disorders, particularly significant hereditary problems in the first trimester. Potential results include stillbirth, congenital toxoplasmosis, or long-lasting serious effects (Abdel-Wahab et al. 2024; Hashemi-Hafshejani et al. 2023).

The standard treatments for acute toxoplasmosis are pyrimethamine and sulfadiazine. Common adverse reactions, such as hematological toxicity, teratogenicity, reduced bone marrow function, and renal problems, severely restrict the use of these drugs (Bahreini et al. 2022; Jafarpour Azami et al. 2021). Spiramycin, an antibiotic from *Streptomyces ambofaciens*, is the primary treatment for *Toxoplasma* infection in expectant mothers. This macrolide antibiotic possesses antiparasitic properties and has been demonstrated to be non-teratogenic (Omar et al. 2021; Tavares et al. 2021). Spiramycin offers considerable advantages, but its ability to cross the blood–brain barrier is restricted, emphasizing the necessity for further research and development (Hagras et al. 2022).

Curcumin (Cur), a naturally occurring phenolic mixture [1,7-bis(4-hydroxy-3-methoxyphenyl)−1,6-heptadien-3,5-dione], is extracted from the underground rhizomes of the perennial plant *Curcuma longa Linnaeus* (Teimouri 2023). Curcumin has therapeutic potential for treating and preventing diseases, including neurological disorders, metal-induced liver harm, diabetes, rheumatoid arthritis, chronic disorder, migraines, and wound healing (Aggarwal and Sung 2009; Bulboacă et al. 2016; Bulboacă et al. 2017; Bulboacă et al. 2018; García-Niño and Pedraza-Chaverrí 2014; Yallapu et al. 2015). However, its greatest limitations are its low water solubility and poor absorption (Azami et al. 2018b; Khezri et al. 2018).

Nanomedicine is a strikingly safe, effective way to deliver herbal combinations. Research has demonstrated that nanomedicine delivery approaches, such as nanoemulsions (NEs), nanocapsules, phytosomes, and nanoliposomes, can enhance the half-life, bioavailability, and pharmacological potency of phytochemicals while simultaneously reducing teratogenicity (Mobaleghol Eslam et al. 2024). Nanoemulsions (NEs) have become a potentially effective method to enhance the oral absorption of drugs that have low solubility in water. These formulations are oil-in-water or water-in-oil emulsions with droplet sizes under 200 nm. They consist of water, oils, and surfactants. The nanoscale droplets enhance clarity and maintain physical stability by preventing noticeable coagulation, precipitation, or phase separation. They also improve absorption efficiency while lowering material use (Firooziyan et al. 2022; Yen et al. 2018).

In our previous work, a spiramycin nanoemulsion (NE-Spi) was prepared and showed promise in inhibiting tachyzoites of *T. gondii*, the RH strain, in vitro (Hashemi-Hafshejani et al. 2023). In the present study, considering that no previous examinations have been performed on the usage of a nanoemulsion containing spiramycin and curcumin for the treatment of toxoplasmosis, this investigation prepared a nanoemulsion containing spiramycin and curcumin for the first time and evaluated its efficacy against both acute and chronic toxoplasmosis in vitro and in vivo.

## Materials and methods

### Compounds

Spiramycin (Rovamycin) was obtained from Merck KGaA in Darmstadt, Germany (Ca No. 8025–81-8), while soybean oil (Ca No. 8001–22-7) and curcumin (Ca No. 458–37-7) were sourced from Sigma–Aldrich Co., based in St. Louis, MO, USA. Polysorbate 80 and 85 were acquired from SRL, India, and ethanol and dimethyl sulfoxide were sourced from Merck Chemicals, Germany. The Razi Vaccine and Serum Research Institute provided the Vero cell line in Karaj, Iran. 3-(4,5-Dimethylthiazol-2-yl)−2,5-diphenyltetrazolium bromide (MTT) was received from Kiazist in Hamadan, Iran. Sigma-Aldrich, USA, supplied Dulbecco’s modified Eagle’s medium (DMEM), fetal bovine serum (FBS), penicillin/streptomycin, propidium iodide, and trypan blue dye.

### NE-CS: Preparation and characterization

Soybean oil, dimethyl sulfoxide (DMSO), a mix of tween 80 and tween 85, and ethanol were utilized as the oil, co-solvent, surfactant, and co-surfactant, respectively. NE-CS, NE-Spi and NE were made via the spontaneous emulsification technique. In brief, for NE-CS formulation, the co-surfactant and distilled water were gradually mixed magnetically with stirring at 100 rpm for an hour. Then, spiramycin and curcumin, which were dissolved in soybean oil and DMSO, were added and mixed (Bouchemal et al. 2004). NE-Spi and NE were prepared by combining spiramycin powder (for NE-Spi), soybean oil, DMSO, Tween 80 and 85. This mixture was then added to the aqueous phase containing distilled water and ethanol, and adequately stirred at room temperature (Hashemi-Hafshejani et al. 2023). The visual monitoring of nanoemulsions for signs of creaming, color alterations, and phase inversion took place over a month-long period. To assess instability, freeze–thaw tests were performed following three consistent cycles. The Brookfield Digital Viscometer and Malvern Zeta Sizer (Nano Z-S; Malvern Instruments, Worcestershire, UK) were utilized to measure zeta potential and particle size. A transmission electron microscope (TEM) model LEO 906, operating at 100 kV, was employed to analyze particle sizes.

### Animals

This research involved female BALB/c mice with a weight range of 20 to 25 g. The mice were maintained in typical situations (12 h light and dark, maintained at around 22 ± 2 °C, and furnished complete access to food and fresh drinking water). The Ethics Committee of Tehran University of Medical Sciences in Tehran, Iran, supported the research on November 18, 2020 (IR.TUMS.SPH.REC.1399.208). All animal experimentations were conducted in accordance with the standards set by the United States National Institutes of Health (NIH) concerning the care and usage of laboratory animals. The methods were approved by the Ethical Committee at Tehran University of Medical Sciences, Tehran, Iran.

### Parasite strains and cell lines

A virulent RH strain (Type I) and an avirulent Tehran strain (Type II) of *T. gondii* were effectively introduced into mice via a series of passages (Ghorbani and Sami 1973). Tachyzoites from the virulent RH strain (Type I) of *T. gondii* were collected from the peritoneal cavity of infected mice, cleaned with PBS (pH: 7.4), and then calculated on a hemocytometer slide. Mice that were infected with the Tehran strain (Type II) of *T. gondii* were euthanized two months after infection. Their brains were extracted and processed using a 22-gauge needle along with 1 ml of PBS. Two drops of 20 µl of the brain homogenate were applied to slides, and the brain cysts were enumerated using a light microscope (Azami et al. 2018b).

### In vitro experiment

#### Cell viability assay

A total of 5 × 10^6^ cells/ml of uninfected Vero cells was cultured in Dulbecco’s modified Eagle’s medium (DMEM) enriched with 10% fetal bovine serum (FBS) and 1% penicillin/streptomycin in cell culture flasks. The culture was kept at 37 °C for 24 h. An MTT assay was completed according to the manufacturer’s teachings to evaluate the viability of uninfected Vero cells at diverse concentrations (1000, 500, 250, 125, 62.5, and 31.25 µg/ml) of the nanoemulsion containing spiramycin and curcumin (NE-CS), spiramycin nanoemulsion (NE-Spi), spiramycin suspension (S-Spi), and a nanoemulsion without the drug (NE).

#### Effects of NE-CS on *T. gondii* tachyzoites

*T. gondii* tachyzoites, the RH strain, were exposed to four various concentrations (250, 125, 62.5, and 31.25 µg/ml) of NE-Spi, NE-CS, S-Spi, and NE within time intervals of 30, 60, 90, and 120 min at room temperature. Additionally, one well was assigned as the infected/untreated (positive) control group. All the investigations were carried out in duplicate. Dead tachyzoites were identified using 0.4% trypan blue dye and counted under a light microscope at 400 × magnification. The percentage of non-viable cells was then calculated to assess the extent of cell death.

#### Flow cytometry

In total, 2 × 10^5^ tachyzoites were subjected to the maximum concentration of 250 μg/ml of NE-CS and maintained at room temperature for 24 h. After that, propidium iodide was added, and the staining procedure took place in the dark for 30 min at 4 °C. The percentage of non-viable tachyzoites was then determined using flow cytometry (FACSCalibur B, BD, USA). The control group consisted of tachyzoites of *T. gondii*, the RH strain, incubated with distilled water under the same conditions as the treated samples. Previous research has examined the flow cytometry of NE-Spi (Hashemi-Hafshejani et al. 2023)**.**

### In vivo experiment

#### Initial assessment of the oral nanoemulsion

For two weeks, three groups consisting of three female BALB/c mice were used. Each mouse received 0.1 mL of NE-CS and NE-Spi daily, corresponding to a dose of 25 mg/kg/day of curcumin, 50 mg/kg/day of spiramycin, and 50 mg/kg/day of NE-Spi, based on average body weight. A control group (uninfected/untreated) of three mice was administered phosphate-buffered saline (PBS). The physical and behavioral traits of the treatment and control groups were observed and documented day-to-day before and after the experiments. After the study, the animals were put down, and blood samples were obtained via intracardiac collection for biochemical analysis. The hepatic and splenic tissues were fixed in 10% formalin, embedded in paraffin, and sliced to a consistency of 5 μm for histological assessment.

#### Treatment of acute toxoplasmosis

Fifty female BALB/c mice were inoculated intraperitoneally with 1000 tachyzoites of the *T. gondii*, the RH strain, and then separated into five groups of ten mice each. Mice in group 1 received 50 mg/kg/day of NE-Spi, while those in group 2 were administered NE-CS (25 mg/kg/day of curcumin and 50 mg/kg/day of spiramycin). Mice in group 3 received 10 mg/kg/day of S-Spi, group 4 was given NE, and group 5 remained infected/untreated, serving as the positive control group. Treatment began three hours post-injection and continued thereafter, and continued daily via oral gavage once a day. Throughout the experimental period, the survival time of each group was monitored and recorded. Beginning from day 5 of the medicine, peritoneal fluid was gathered from five mice in each group to assess the parasite load and growth by counting tachyzoites under light microscopy at 400 × magnification. To assess the virulence of tachyzoites post-treatment, suspensions of the parasites were administered to new sets of mice from each group (*n* = 6), and the survival time was recorded.

#### Treatment of chronic toxoplasmosis

Tissue cysts from the Tehran strain of *T. gondii* were introduced into fifty female BALB/c mice through an intraperitoneal injection of a brain suspension from an infected mouse containing 600 tissue cysts. Two weeks after infection, the mice were categorized into five groups, following the same approach used in the acute phase study. The study continued for 14 days daily via oral gavage once a day. Subsequently, the mice were put down, and their brain tissue was collected to investigate the presence of tissue cysts. Squash smears were prepared from the brain tissues of the mice; all brain tissue cysts present in each sample were counted and measured under light microscopy (400 × magnification) using a calibrated ocular micrometer. A suspension of tissue cysts from the initial cohort was subsequently injected into the peritoneal cavity of another group of mice (*n* = 5). The tissue cyst suspension from the initial group was injected into the peritoneum of the mice (*n* = 5). Mice were euthanized four weeks later, and then the cysts present in the brain tissues were estimated.

### Statistical analysis

The results were analyzed with SPSS (version 22; IBM Corp., Armonk, NY, USA) and GraphPad Prism 9.0. A statistical value point was set at a *p*—value of less than 0.001. The survival rate of the mice was evaluated using the Kaplan–Meier method.

## Results

### Preparation of NE-CS

**Fig. 1:**
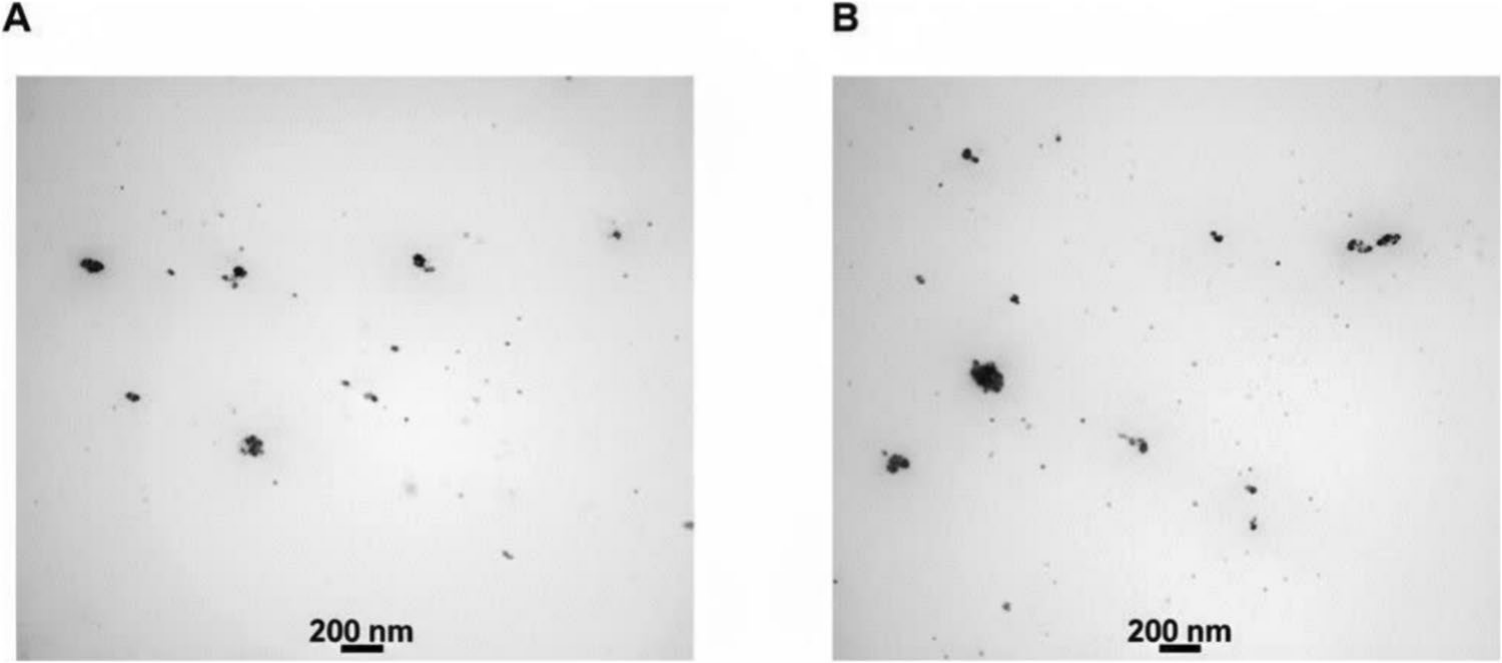
Transmission electron micrograph of NE-CS (**A**), NE (**B**)

In the oily phase, soybean oil (6%), DMSO (16%), tween 80 (24%), tween 85 (14%), spiramycin powder (1%), and curcumin (0.5%) were dissolved. The mixture was subsequently combined with the aqueous phase, which included 10% ethanol and distilled water. As previously described, to prepare the spiramycin nanoemulsion, spiramycin powder (1%), DMSO (5%), soybean oil (5%), tween 80 (24%), and tween 85 (14%) were used (Hashemi-Hafshejani et al. 2023), and for the NE, soybean oil (5%), tween 80 (24%), and tween 85 (12%) were used. This mixture was then added to the aqueous phase containing ethanol (10%) and distilled water, and adequately stirred at room temperature. The particle size, determined by DLS, was 13.9, 10.4, and 12.2 nm for the NE-CS, NE-Spi, and NE, respectively, and the measured zeta potentials were −28.3, −33.7, and −20.2 mV for the NE-CS, NE-Spi, and NE, respectively. The stability data over one month revealed no phase separation, cloudiness, or color changes for NE-CS, NE-Spi, and NE. Moreover, following freezing and thawing cycles, no noticeable changes in particle size were observed (NE-CS: 15.8 nm, NE-Spi: 11.3 nm, and NE: 14.6 nm). TEM was also used to verify the spherical form and size of the nanoemulsion particles (Fig. 1). Detailed characterization of the NE-Spi formulation, including DLS, zeta potential, and TEM-based morphology, has been previously published (Hashemi-Hafshejani et al. 2023).

### In vitro experiments

#### Cell viability assay

**Fig. 2:**
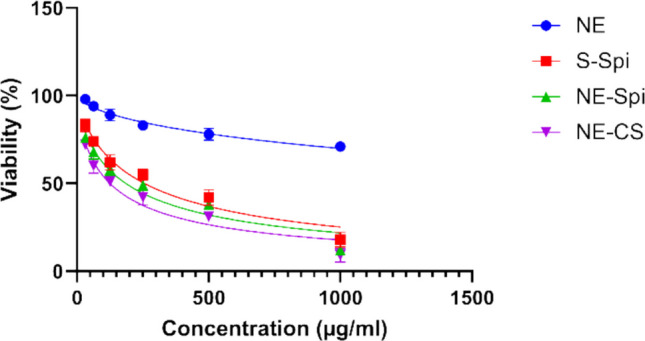
Assessment of cell viability via MTT assays. Uninfected Vero cells were treated with NE-Spi, NE-CS, S-Spi, and NE at different concentrations (1000, 500, 250, 125, 62.5, and 31.25 µg/ml)

The MTT assay revealed that at the highest concentration of NE-CS (1000 µg/ml), approximately 9% of the uninfected Vero cells cells remained viable. As the NE-CS concentration decreased, cell viability improved, reaching up to 72% at the lower levels tested. Also, 12% and 18% of uninfected Vero cells were viable with NE-Spi and S-Spi, respectively, at a 1000 µg/ml concentration. Figure 2 shows the impacts of NE-Spi, NE-CS, S-Spi, and NE on uninfected Vero cells at six doses.

#### Effects of NE-CS on tachyzoites of the *T. gondii*, RH strain

**Fig. 3:**
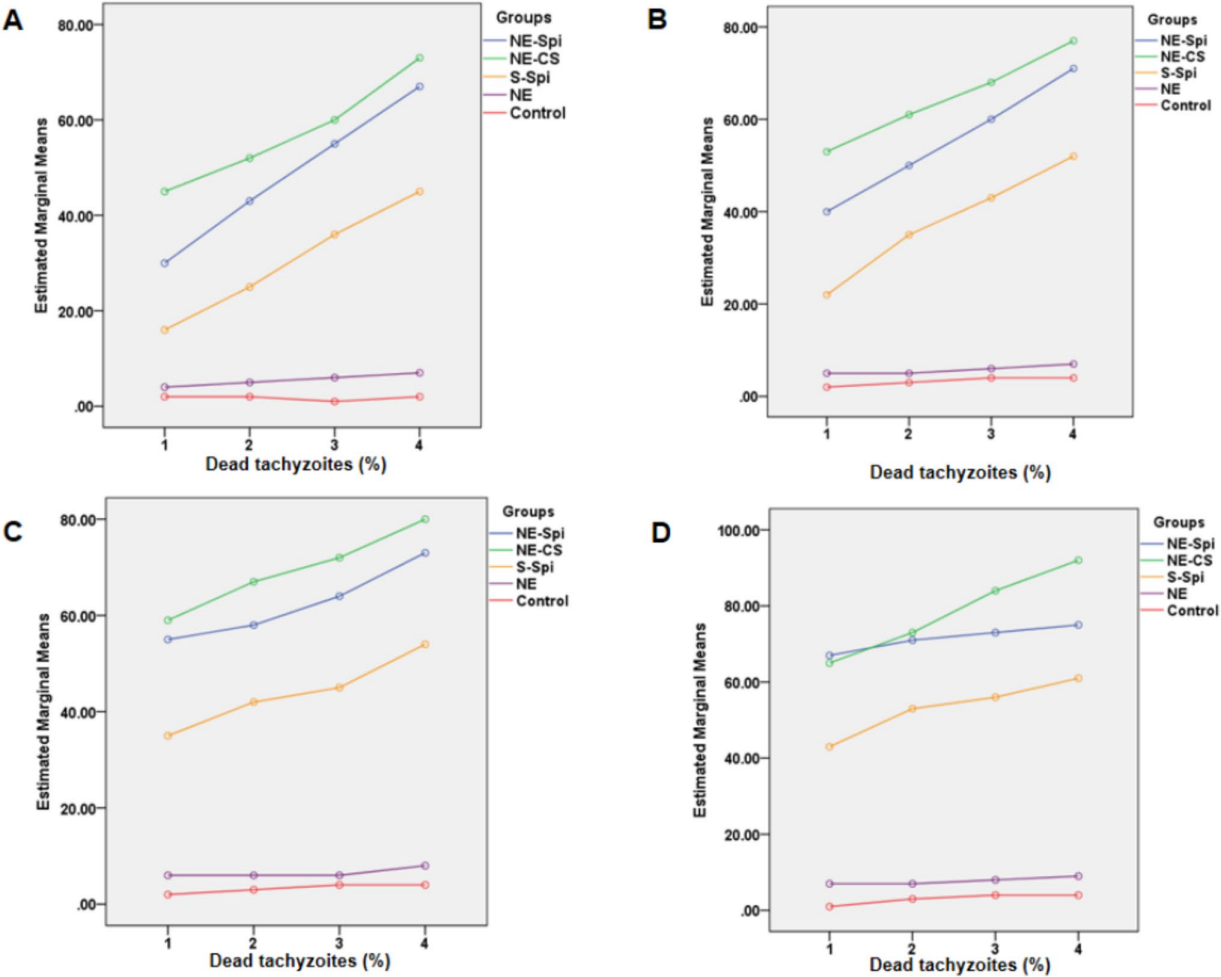
Comparative in vitro evaluation of the effects of NE-Spi, NE-CS, S-Spi, and NE concentrations of 31.25 (**A**), 62.5 (**B**), 125 (**C**), and 250 (**D**) μg/ml on tachyzoites of *T. gondii*, the RH strain, in comparison with the control group

The results indicated that NE-Spi, NE-CS, S-Spi, and NE at concentrations of 31.25, 62.5, 125, and 250 µg/ml were effective in reducing tachyzoites of the *T. gondii*, the RH strain, following 30, 60, 90, and 120 min of exposure. A statistically meaningful difference in the mean percentage of dead tachyzoites was observed among the treatment groups, as determined by one-way ANOVA (*p* < 0.001). In a pairwise comparison of groups via the POST HOC test (Bonferroni), the difference between the NE-CS group and the S-Spi and NE and control groups was significant (*p* < 0.001). Conversely, no notable difference was found between the NE-CS and the NE-Spi groups (*p* = 0.25). Figure 3 shows the outcomes of treating tachyzoites of *T. gondii*, the RH strain, with NE-Spi, NE-CS, S-Spi, NE, and the control.

#### Flow cytometry

**Fig. 4:**
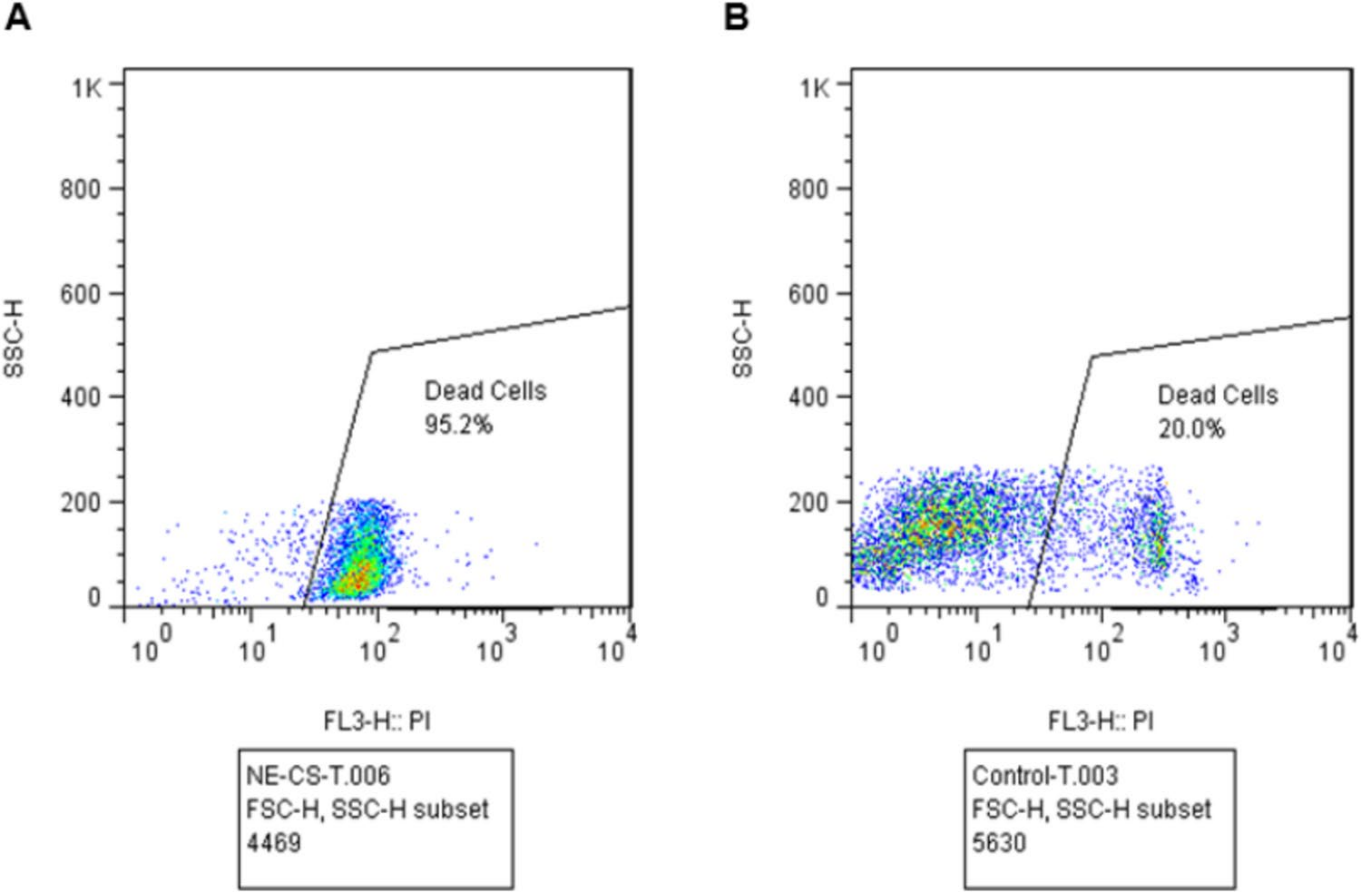
Flow cytometry results at 250 µg/ml concentration of NE-CS on tachyzoites of *T. gondii*, the RH strain after exposure for 24 h (**A**) compared with the control group (consisted of tachyzoites of *T. gondii*, the RH strain, incubated with distilled water) (**B**)

Flow cytometry was utilized to compare the death percentage of tachyzoites of the *T. gondii*, the RH strain, 24 h after exposure to NE-CS (250 μg/ml) with those of the control group (consisted of tachyzoites of *T. gondii*, the RH strain, incubated with distilled water). Figure 4 displays the findings. At a concentration of 250 μg/ml NE-CS, the most significant death percentage of 95.2% was observed.

### In vivo experiments

#### Toxicity studies

**Fig. 5:**
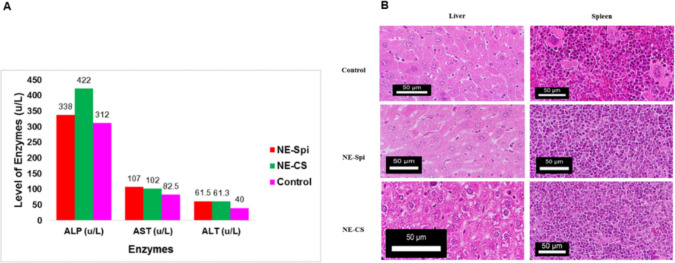
Serum levels of AST, ALT, and ALP in the mice in the NE-Spi and NE-CS treatment groups and the control group (**A**). Representative histological images of the spleen and liver from the treated and control groups (**B**)

Nine female BALB/c mice were divided into NE-Spi-treated, NE-CS-treated, and control mice (uninfected/untreated). Throughout the experiment, all the mice in the three groups survived with no observable adverse clinical signs of toxicity (e.g., decreased activity, hair loss, weight loss, or abnormal feeding and drinking). Histopathological analysis of the liver and spleen tissues demonstrated no notable pathological modifications in the treated groups in comparison to the control group (Fig. 5B). In addition, serum biochemistry analysis did not show any significant differences in the values of alanine aminotransferase (ALT), aspartate aminotransferase (AST), and alkaline phosphatase (ALP) for NE-Spi, NE-CS, and control groups (Fig. 5A).

#### Acute studies

**Fig. 6:**
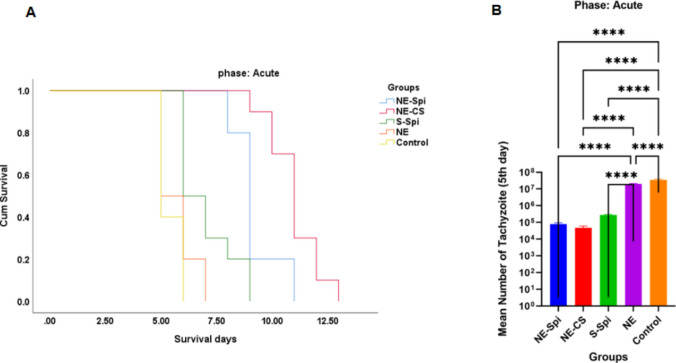
The effectiveness of several compounds against *T. gondii* in female BALB/c mice contaminated with tachyzoites of *T. gondii*, the RH strain, as well as the potential virulence of the tachyzoites after treatment. Survival time of inoculated mice with tachyzoites of *T. gondii*, the RH strain, and those treated with NE-CS, NE-Spi, S-Spi, and NE compared with the positive control group (*p* < 0.001) (**A**), The mean number of peritoneal tachyzoites of mice treated with NE-Spi, NE-CS, S-Spi, and NE and the positive control group without treatment on the fifth day of infection (*p* < 0.001) Mean ± SD (**B**)

The Kaplan‒Meier test was utilized to measure the survival time of each treatment group (Fig. 6A). The mice that received NE-CS began dying 9 days after treatment and survived until day 13. In contrast, deaths in the untreated group started on day 5, with all the mice dying by day 6. The mice that received NE-Spi, S-Spi, and NE began to die on days 8, 5, and 5, respectively, with survival times of 11, 9, and 7 days. All treatment groups exhibited longer survival times in comparison to the positive control group (infected/untreated), and there were significantly different survival times noted in the mice treated with NE-CS compared to the positive control group (*p* < 0.001). On the fifth day post-infection, a meaningful difference (*p* < 0.001) in the average counts of peritoneal tachyzoites was noted across all groups, as assessed by one-way analysis of variance. NE-CS markedly reduced the intracellular multiplication of *T. gondii* tachyzoites (5 ± 2.78 × 10^4^) in comparison to the positive control group (3509 ± 435.39 × 10^4^). The number of peritoneal tachyzoites in the groups treated with NE-Spi, S-Spi, and NE was 8 ± 1.49 × 10^4^, 28 ± 2.49 × 10^4^ and 2000 ± 115.82 × 10^4^, respectively. Compared with the S-Spi, NE, and positive control treatments, treatment with NE-CS and NE-Spi significantly decreased the growth of tachyzoites (Fig. 6B).

**Fig. 7:**
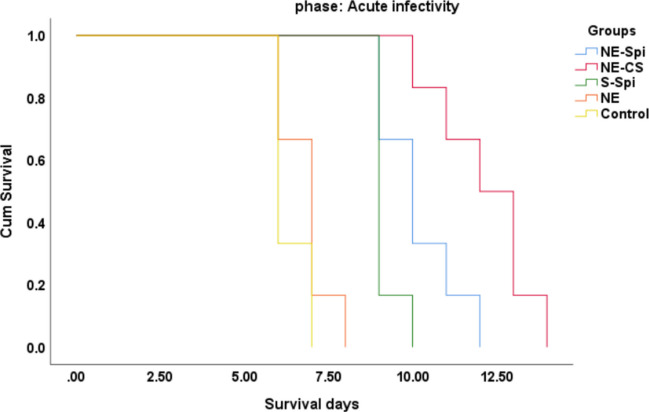
Survival time of group 2 of mice inoculated with tachyzoites of *T. gondii*, the RH strain, acquired from group 1, that were treated with NE-Spi, NE-CS, S-Spi, and NE, compared with those in the positive control group

After new groups of mice were inoculated with tachyzoites received from the initial group, the survival rate was determined. The results showed that mice receiving tachyzoites from NE-Spi and NE-CS-treated mice had longer survival times (Fig. 7). After injecting tachyzoites from the initial group, the mean survival periods of the mice treated with NE-Spi and NE-CS were 12 and 14 days, respectively.

#### Chronic studies

**Fig. 8:**
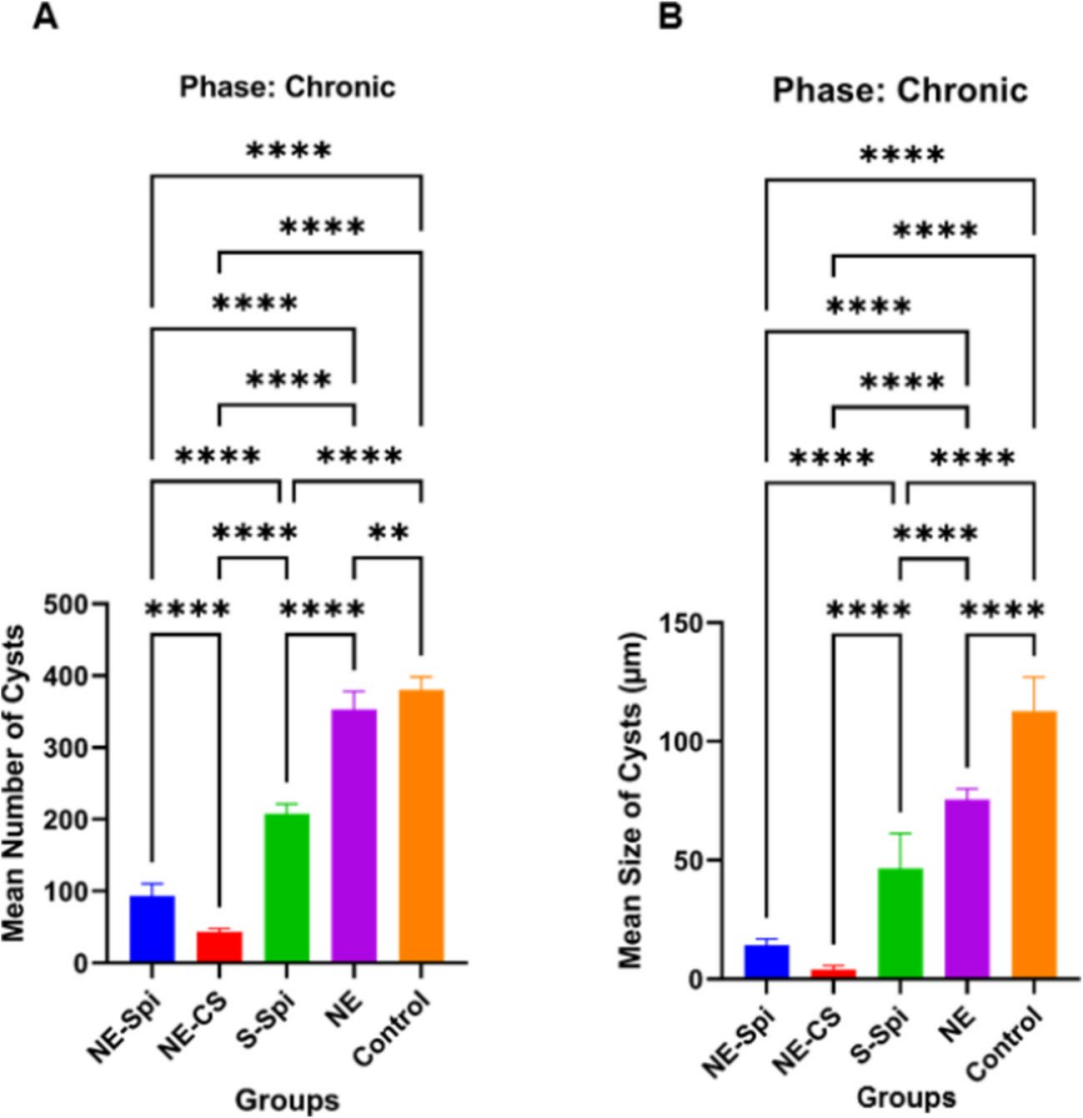
Mean number (**A**) and size (**B**) ± SD of tissue cysts in mice inoculated with bradyzoites of *T. gondii*, Tehran strain, treated with NE-CS, NE-Spi, S-Spi, or NE compared with those in the positive control group (*p* < 0.001)

As shown in Fig. 8, the average number and size of brain tissue cysts in the treatment groups were considerably lower than in the positive control group (infected/untreated) (*p* < 0.001). In the mice administered NE-CS treatment, the mean number and size of brain tissue cysts were measured at 43 ± 5.78 and 4 ± 1.11 µm, respectively. The counts and dimensions of tissue cysts in the mice treated with NE-Spi were 93 ± 8.15 and 14.3 ± 3.31 µm, while the values for mice treated with S-Spi were 207 ± 12.38 and 51.6 ± 5.62 μm. For those receiving NE treatment, the figures were 353 ± 15.71 and 75.6 ± 6.32 μm, compared to 380 ± 17.22 and 112.8 ± 8.28 μm for the infected/untreated mice.

**Fig. 9:**
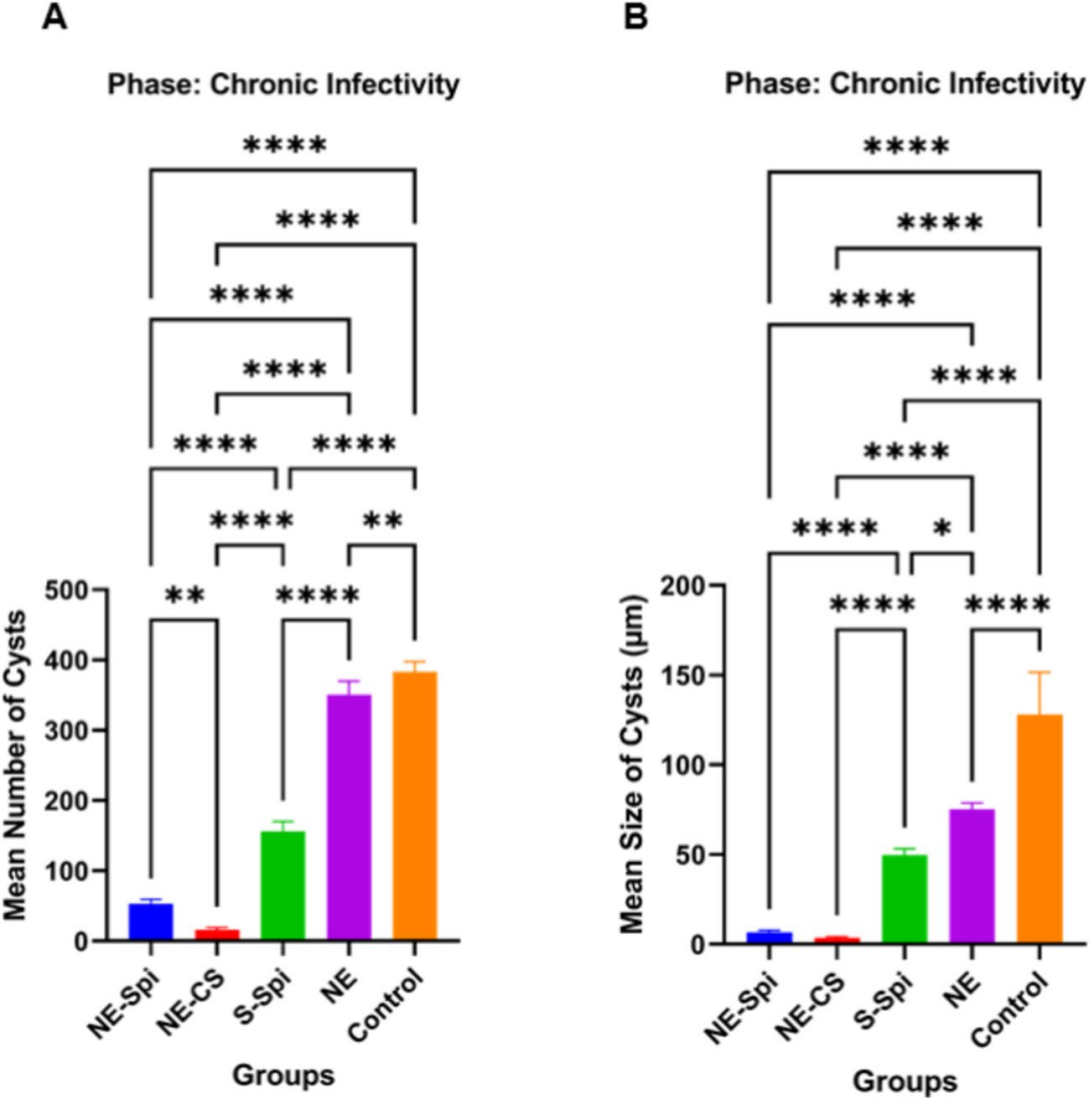
Mean number (**A**) and size (**B**) of tissue cysts in the second group of mice inoculated with bradyzoites of *T. gondii*, Tehran strain, from the first group that were treated with NE-CS, NE-Spi, S-Spi, and NE compared with those in the positive control group (*p* < 0.001)

Following the inoculation of brain suspensions from the initial group of mice into the second group, meaningful statistical differences were noted in both the number and size of cysts (Fig. 9A and Fig. 9B) when comparing the control group that received untreated brain suspensions with the group that received treated brain suspensions, especially those treated with NE-CS (*p* < 0.001).

## Discussion

*T. gondii* is among the most infectious parasites worldwide, capable of infecting a broad range of hosts. This parasite infects almost half of the human population and often causes no symptoms. This parasite’s tachyzoites can cross the blood‒brain barrier, resulting in lethal encephalitis, presenting a significant challenge for researchers in therapeutic efforts. In this context, the search for innovative therapy protocols should be constantly monitored to identify effective and well-tolerated treatments (Allam et al. 2022; Wang et al. 2011).

An effective pharmacological agent for toxoplasmosis management would exhibit anti-parasitic efficacy against several parasitic stages. It should also have efficient tissue penetration. Numerous therapeutic medications are utilized; however, they may be associated with various and extreme harmful effects (FarahatAllam et al. 2020). Spiramycin is a macrolide antibiotic used safely to treat *T. gondii* during pregnancy (Abdel Hamed et al. 2019). Despite the remarkable advantages of spiramycin, it results in limited penetration of the blood‒brain barrier. Therefore, further development of spiramycin is essential to capitalize on its benefits (Allam et al. 2022; Chew et al. 2012; Hagras et al. 2019). Reviews indicate that the herbs used for medicinal purposes in conventional medicine include a range of substances with diverse biological and therapeutic properties, particularly in managing microbial infections (Shaapan et al. 2021). Several studies have focused on combining natural substances with well-established pharmaceuticals to increase their biological efficacy (Mendez-Pfeiffer et al. 2021). Data regarding the efficacy of spiramycin-loaded curcumin nanoemulsion (NE-CS) against toxoplasmosis is lacking despite the evaluation of numerous nanoformulations of spiramycin and other existing pharmaceuticals in vivo or in vitro (Albalawi et al. 2021; El-Zawawy et al. 2015; El Temsahy et al. 2016; Etewa et al. 2018). This research examined the medicinal effects of NE-CS on virulent and avirulent *T. gondii* strains in vitro and in a mouse model, and showed promise in reducing parasite burden and enhancing survival. The generated nanoemulsions were assessed via dynamic light scattering (DLS) particle size measurements, transmission electron microscopy (TEM) morphology and particle size estimation, zeta potential, and stability studies (Azami et al. 2018a; Azami et al. 2018b; Teimouri et al. 2018). In the present study, the particle size was determined via dynamic light scattering (DLS) and confirmed to be within the nanometer range. The particle size was 15.8 nm, as measured by DLS, and the zeta potential was −28.3 mV for NE-CS. TEM was also used to verify the spherical form and size of the nanoemulsion particles. The in vivo toxicity of NE-CS showed no behavioral or physical signs of toxicity in the treated mice. Furthermore, histological and biochemical analyses revealed no marked anomalies in the liver and spleen tissues or the liver enzymes of the animals. These findings confirmed the feasibility of using nanoemulsions as a safe drug delivery technology (Azami et al. 2018a; Azami et al. 2018b). Our findings corroborated prior research demonstrating the favorable safety of nanoformulations both in vitro and in vivo (Khosravi et al. 2020; Schöler et al. 2001; Sood et al. 2014).

The studies on survival time revealed that the mean survival time for mice treated with NE-CS exceeded that of the positive control group, indicating enhanced therapeutic effectiveness against acute toxoplasmosis. Additionally, the treated mice exhibited a reduced average number of peritoneal tachyzoites compared to the control group (infected/untreated). In acute experimental toxoplasmosis, mice administered NE-CS experienced the longest average survival time, reaching a maximum of 13 days. This demonstrates that NE-CS is more effective against acute toxoplasmosis. The tachyzoite count in peritoneal exudates significantly reduced for both the NE-CS and NE-Spi groups. It is worth noting that the NE-CS group received a higher total drug dose (spiramycin + curcumin), which may have contributed to the enhanced therapeutic effect observed. Therefore, the superior outcomes in this group may reflect both the benefits of nanoformulation and the increased drug concentration. This finding is consistent with recent investigations, including those by Azami et al., Goudarzi et al., and Siahkal et al., which have demonstrated that drug-loaded nanoparticles can prolong survival and decrease parasite burden in mice infected with the *T. gondii*, the RH strain (Azami et al. 2018a, b; Goudarzi et al. 2024; Siahkal et al. 2023). The impact of the curcumin nanoemulsion and suspension on the survival time and tachyzoite numbering in the peritoneal fluid of mice infected with tachyzoites of the *T. gondii*, the RH strain, during the acute phase was investigated in a study by Azami SJ et al. under comparable conditions; the results revealed that mice treated with the curcumin nanoemulsion for 8–10 days after the treatment survived, and the number of tachyzoites identified in the peritoneal fluid was determined to be 627.5 ± 73 × 10^4^. The number of surviving days for mice whose curcumin suspension was administered was between 5 and 8 days, and the number of tachyzoites found in the peritoneal fluid was 4745 ± 340 × 10^4^ (Azami et al. 2018b). A comparable substantial decrease was observed in animals administered atovaquone-loaded macrophage-derived exosomes (Goudarzi et al. 2024) and in those receiving zinc oxide nanoparticles (ZnO-NPs) (Siahkal et al. 2023). Hagras et al. (2019) investigated spiramycin-loaded chitosan nanoparticles against *Toxoplasma* and reported a substantial decrease in parasite count in infected mice and increased survival duration (Hagras et al. 2019). Teimouri et al. (2018) evaluated the effectiveness of various molecular weights and concentrations of chitosan against *Toxoplasma* and found significant anti-*Toxoplasma* toxicity in all parameters they tested (Teimouri et al. 2018). Etewa et al. (2018) evaluated spiramycin-loaded chitosan nanoparticles for the treating of chronic and acute toxoplasmosis and reported that this treatment was toxic to both *Toxoplasma* tachyzoites and brain cysts (Etewa et al. 2018). In contrast to the other groups, the NE-CS-treated group showed significant decreases in both the number and average size of brain cysts in mice with chronic *T. gondii* infection from the Tehran strain. A study performed under similar conditions by Azami SJ et al. revealed that, compared with a control, curcumin nanoemulsion (CR-NE) and suspension (CR-S) significantly reduced the number and size of brain tissue cysts in *T. gondii*-infected mice (Tehran strain) (*p* < 0.001) (Azami et al. 2018b). In the research by Abdel-Wahab AA et al. on toxoplasmosis treatment, the group that received spiramycin-loaded maltodextrin nanoparticles showed a notable decrease in brain cyst number compared to the other subgroups (Abdel-Wahab et al. 2024).

The anti-*Toxoplasma* effects of curcumin may come from several mechanisms. Studies have shown that curcumin boosts the host’s immune responses by increasing proinflammatory cytokines like IFN-γ and IL-1β, which are important for controlling parasites. It also lowers oxidative stress and inflammation by adjusting the levels of antioxidant enzymes. Curcumin induces apoptosis and necrosis in tachyzoites and increases nitric oxide production, contributing to its direct antiparasitic activity (Ezzatkhah et al. 2023). Furthermore, in neural models, curcumin modulates purinergic receptors (A1, A2A, P2X7), which are involved in neuroinflammatory regulation and parasite clearance (Bissacotti et al. 2023). These multifaceted actions support the observed efficacy of NE-CS in both acute and chronic phases of toxoplasmosis.

In addition to enhancing the effectiveness of the active component, the antioxidant and antibacterial properties of soybean oil may contribute to the decrease in both the quantity and size of cysts in mice treated with NE-CS compared to those in the positive control group (infected/untreated) during chronic trials. Additionally, the treatments’ effects on the pathogenic ability of the RH and Tehran strains of *T. gondii* were analyzed. Tachyzoites and tissue cysts from both the treated and untreated groups were analyzed. The findings indicated that treatment with NE-CS and NE-Spi diminished the virulence of the parasite. In comparison to nanoemulsified spiramycin, the nanoemulsion containing both spiramycin and curcumin exhibited greater therapeutic effectiveness. It is important to note that the reduced efficacy of S-Spi compared with NE-Spi may be attributed not only to the difference in formulation but also to the lower dose administered in the S-Spi group, which was chosen based on solubility and tolerability limitations.

Although these promising results exist, this research has certain limitations. The experiments were done only in mice, which may not fully reflect the complexity of human toxoplasmosis. We still need to establish clinical relevance. Future studies should validate our results in larger animal models and in clinical trials. While the sample sizes were sufficient for initial analysis, they may limit the broad applicability of the results.

Another limitation of the present study is the absence of pharmacokinetic data to directly confirm enhanced bioavailability and brain delivery of the nanoemulsion formulation. Although direct pharmacokinetic data were not collected, the observed therapeutic improvements suggest enhanced bioavailability and brain delivery, warranting further PK studies to confirm this hypothesis. However, therapeutic outcomes suggest improved efficacy; future studies should include comparative PK analyses of NE-CS and NE-Spi, particularly measuring drug concentrations in plasma and brain tissue, to validate this hypothesis.

While the NE-CS formulation showed promising therapeutic effect versus acute and chronic toxoplasmosis, there are several areas to consider for the future. Drug loading and encapsulation efficiency of spiramycin and curcumin were not measured, which can be a more complete indication of the delivery profile and can help to further optimize therapeutic performance. In addition, in vitro drug release under physiological conditions was not quantified, which restrained the comprehension of the extended release profile and mechanism of action. Release kinetics studies and comprehensive physicochemical characterizations must be encompassed in future studies to improve the translational value of the formulation.

We must assess NE-CS’s long-term safety, the best dose, and its pharmacokinetics. We should also assess side effects and how they perform with sufficient size in patient groups. We did not assess the host immune response, which can greatly affect efficacy.

## Conclusions

This research demonstrated that NE-CS exhibits measurable anti-*Toxoplasma* effects in both in vitro and murine models of both acute and chronic infections. In vitro, NE-CS at the highest concentration (250 µg/ml) and longest exposure time (120 min) resulted in a 92% reduction of tachyzoites of the *T. gondii*, the RH strain. In vivo, NE-CS (25 mg/kg/day of curcumin and 50 mg/kg/day of spiramycin) was well tolerated and effectively reduced the parasite load (5 ± 2.78 × 10^4^) compared with that of the positive control group (infected/untreated) (3509 ± 435.39 × 10^4^), improving survival time (up to 13 days in NE-CS treated mice) and a reduction in both number (43 ± 5.78) and size (4 ± 1.11 µm) of brain tissue cysts in the Tehran strain chronic model. These findings emphasize the therapeutic potential of NE-CS and support its further research to explore long-term efficacy, underlying mechanisms, and translational relevance to human toxoplasmosis.

## Data Availability

The data that support the findings of this study are available from the corresponding author upon reasonable request.

## Abbreviations

NE-CS: Nanoemulsion containing spiramycin and curcumin
NE-Spi: Spiramycin nanoemulsion
S-Spi: Spiramycin suspension
NE: Nanoemulsion without the drug
*T. gondii*: *Toxoplasma gondii*
DMSO: Dimethyl sulfoxide
MTT: 3-(4, 5-Dimethylthiazol-2-yl)-2,5-diphenyltetrazolium bromide
DMEM: Dulbecco’s modified Eagle’s medium
FBS: Fetal bovine serum
TEM: Transmission electron microscope
ALT: Alanine aminotransferase
ALP: Alkaline phosphatase
AST: Aspartate aminotransferase
